# Supplementary figures and images for: TRIM25 targets p300 for degradation
Source: Life Sci Alliance. 2023 Sep 28;6(12):e202301980. doi: 10.26508/lsa.202301980 (PMC10539465; doi:10.26508/lsa.202301980)

Figure 1A.I

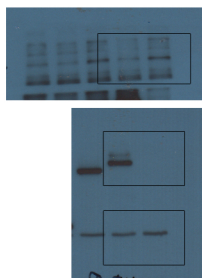

Figure 1A.II

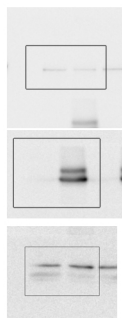

Figure 1A.III

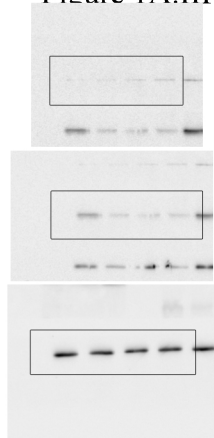

Figure 1B

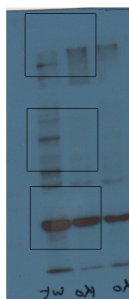

Figure 1C

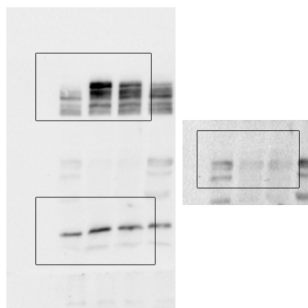

Figure 1E

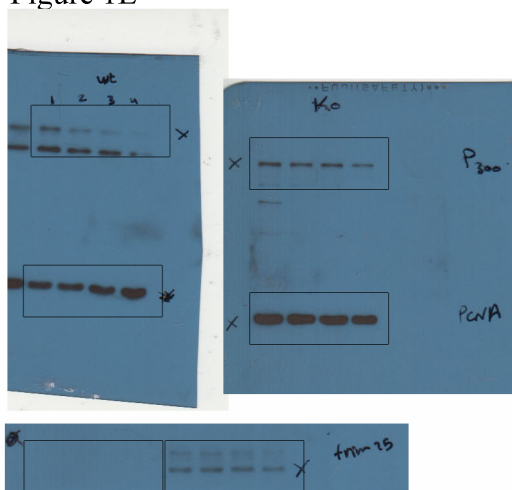

Supplement: Supplementary file 1 [file LSA-2023-01980_SdataF1.pdf]

Figure S1 A

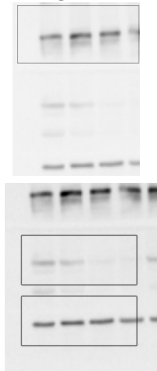

Figure S1 B.I

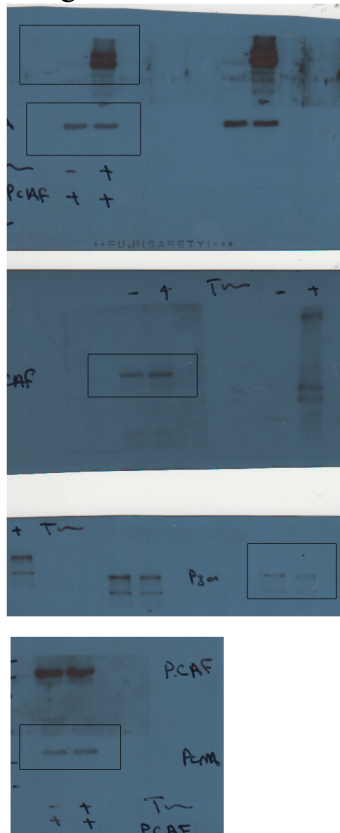

Figure S1 B.II

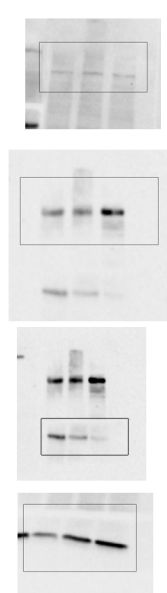

Figure S1 B.IV

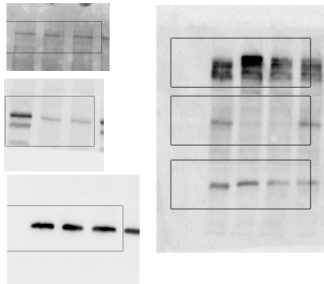

Supplement: Supplementary file 2 [file LSA-2023-01980_SdataFS1.pdf]

Figure 2A

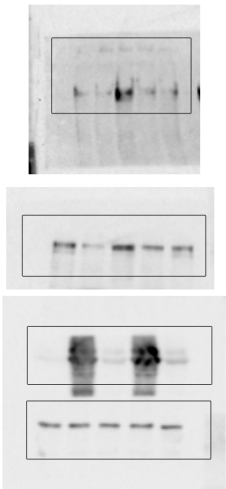

Figure 2B

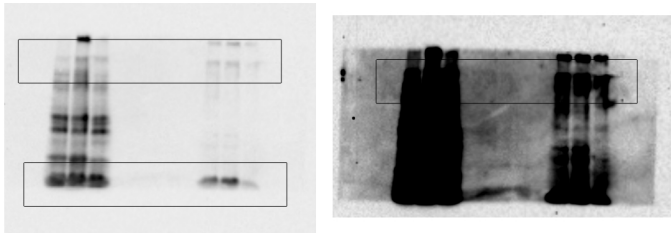

Figure 2C

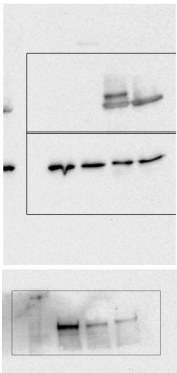

Figure 2D

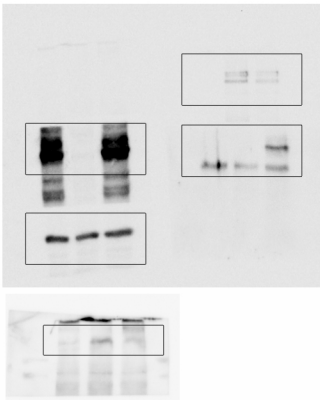

Supplement: Supplementary file 3 [file LSA-2023-01980_SdataF2.pdf]

Figure S3A-F

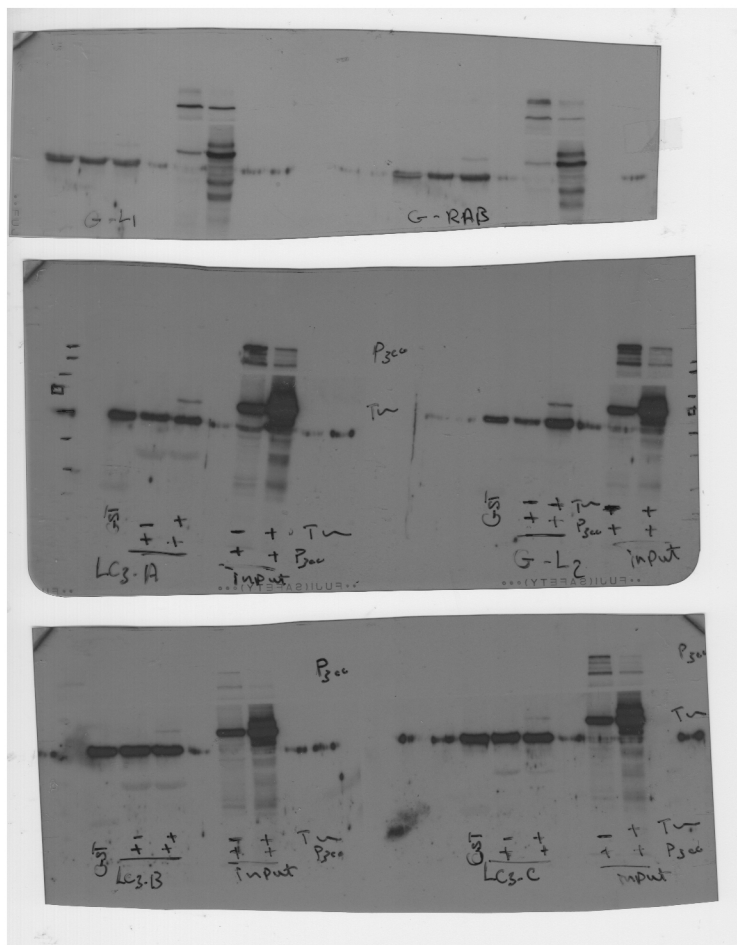

Figure S3G

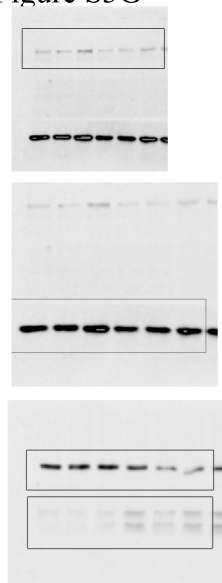

Supplement: Supplementary file 4 [file LSA-2023-01980_SdataFS3.pdf]

Figure 3A

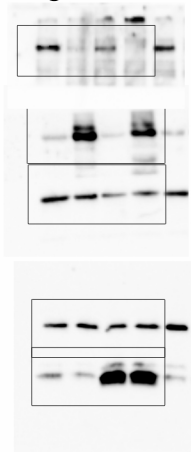

Figure 3B

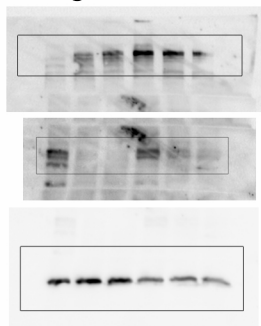

Figure 3C

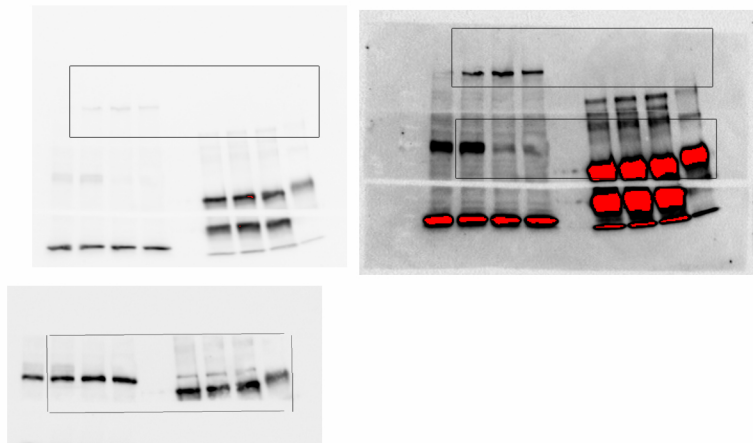

Figure 3D.I

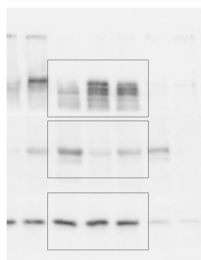

Figure 3D.II

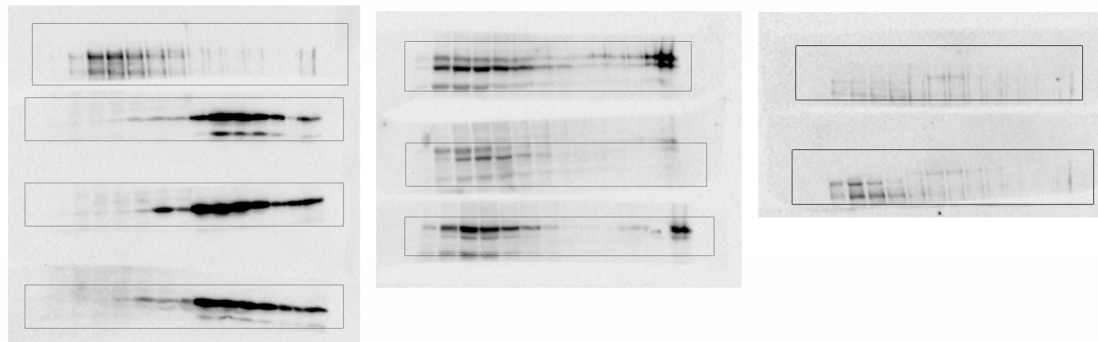

Figure 3E

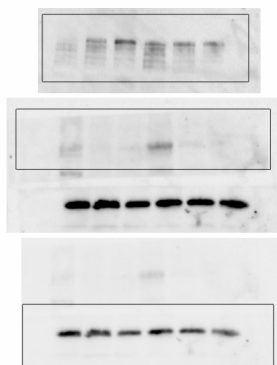

Figure 3F

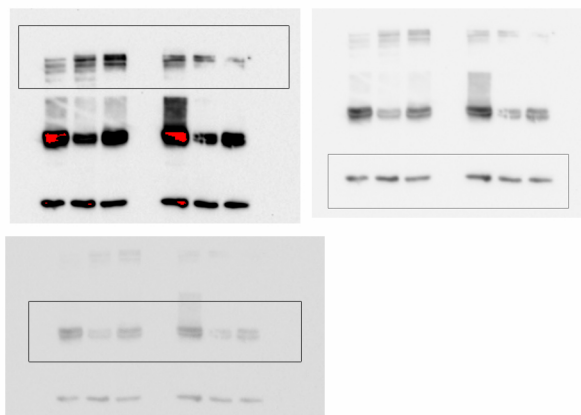

Figure 3G

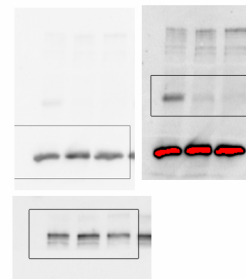

Supplement: Supplementary file 5 [file LSA-2023-01980_SdataF3.pdf]

Figure S5A

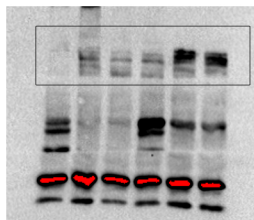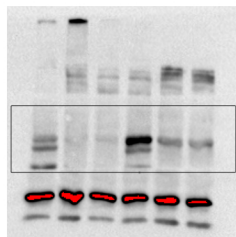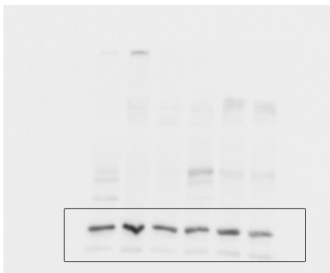

Figure S5B

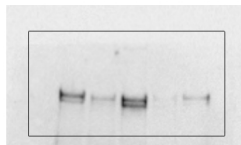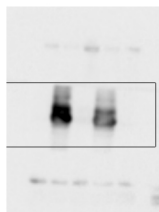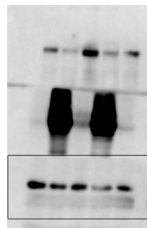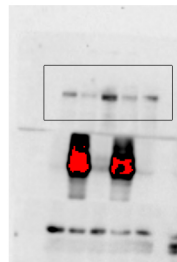

Supplement: Supplementary file 7 [file LSA-2023-01980_SdataFS5.pdf]

Figure 5A

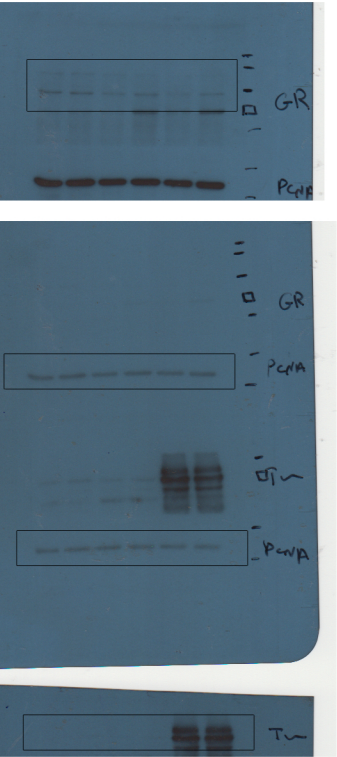

Figure 5B

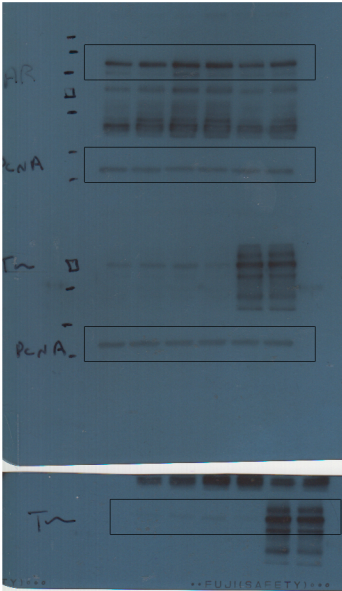

Supplement: Supplementary file 10 [file LSA-2023-01980_SdataF5.pdf]

supplementary figure7D

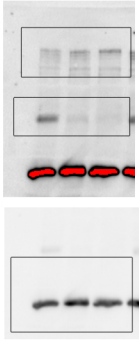

Supplement: Supplementary file 11 [file LSA-2023-01980_SdataFS7.pdf]
