# Supplementary material for: TRIM25 targets p300 for degradation
Source: Life Sci Alliance. 2023 Sep 28;6(12):e202301980. doi: 10.26508/lsa.202301980 (PMC10539465; doi:10.26508/lsa.202301980)
Supplement: Supplementary file 6 [file LSA-2023-01980_SdataFS4.pdf]

Figure S4A

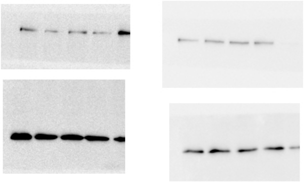

				p300	PCNA	p300/PCNA	%		E1	E2	E3	MV	STD
DMSO	wt	N1	0	4.834.083	17.689.033	0,27328136	100		100	100	100	100	0
			2	1.925.134	14.495.740	0,13280688	48,5971234		48,5971234	129,363553	54,0662909	77,3423225	45,1346241
			4	3.728.861	14.907.912	0,25012631	91,5270286		91,5270286	100,476015	31,4721356	74,4917262	37,5237921
			6	2.360.912	13.395.690	0,17624415	64,49183		64,49183	61,8481029	31,5784002	52,6394444	18,2872362
	wt	N2	0	12.383.447	8.454.255	1,46475911	100						
			2	13.472.740	7.110.134	1,89486443	129,363553						
			4	12.283.447	8.346.255	1,47173157	100,476015						
			6	8.297.669	9.159.326	0,90592572	61,8481029						
	wt	N3	0	10.885.447	3.890.376	2,79804497	100						
			2	13.802.569	9.123.861	1,51279913	54,0662909						
			4	7.894.497	8.964.861	0,88060451	31,4721356						
			6	7.687.326	8.700.225	0,88357784	31,5784002						
EPO	wt	N1	0	8.572.740	11.686.205	0,73357775	100		E1	E2	E3	MV	STD
			2	12.055.690	14.756.569	0,81697107	111,368027		100	100	100	100	0
			4	12.703.326	12.233.154	1,03843424	141,557488		111,368027	191,141091	96,4820222	132,997047	50,9013328
			6	12.288.154	12.597.619	0,97543464	132,969496		141,557488	128,774145	97,1184788	122,483371	22,877648
									132,969496	159,475141	219,277958	170,574199	44,211757
	wt	N2	0	13.209.861	10.514.276	1,25637381	100						
			2	11.387.326	4.741.861	2,40144661	191,141091						
			4	12.904.497	7.976.154	1,61788463	128,774145						
			6	16.294.447	8.132.569	2,0036039	159,475141						
	wt	N3	0	5.770.569	21.951.844	0,262874	100						
			2	9.020.861	35.567.551	0,25362615	96,4820222						
			4	6.744.326	26.417.338	0,25529923	97,1184788						
			6	7.436.033	12.900.267	0,57642474	219,277958						

Figure S4B

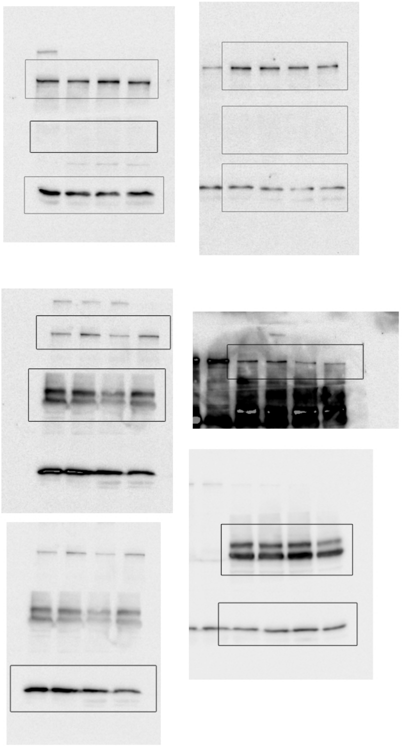

			p300	PCNA	p300/PCNA	%		E1	E2	E3	E4	MV	STD
wt+DMSO	E1	0	25.902.945	24.833.066	1,04308284	100	0	100	100	100		100	0
		2	28.427.208	33.321.823	0,85311083	81,7874473	2	81,7874473	42,1032551	52,5982182		58,8296402	20,5628729
		4	15.867.723	40.462.338	0,39216031	37,5962766	4	37,5962766	24,3972813	32,5562202		31,5165927	6,6606297
		6	3.745.903	28.776.874	0,1301706	12,4794117	6	12,4794117	13,2267754	14,4794789		13,395222	1,01061762
	E2	0	29.932.309	13.138.803	2,27816103	100							
		2	30.226.187	31.512.530	0,95917995	42,1032551							
		4	16.465.945	29.625.167	0,55580936	24,3972813							
		6	10.661.066	35.380.359	0,30132724	13,2267754							
	E3	0	64.938.593	34.715.794	1,87057778	100							
		2	29.429.167	29.911.016	0,98389058	52,5982182							
		4	25.620.238	42.070.087	0,60898942	32,5562202							
		6	9.905.924	36.573.480	0,27084992	14,4794789							
wt+EPO	E1	0	25.790.551	50.182.945	0,5139306	100		E1	E2	E3	E4	MV	STD
		2	35.439.693	56.824.673	0,62366735	121,352445	0	100	100	100		100	0
		4	14.252.773	37.001.309	0,38519645	74,9510642	2	121,352445	187,293422	153,337733		153,994533	32,9753947
		6	30.791.359	22.537.137	1,3662498	265,843249	4	74,9510642	189,072055	89,5129339		117,845351	62,1123538
							6	265,843249	45,0851583	162,868953		157,932454	110,461805
	E2	0	33.004.823	32.132.480	1,02714832	100							
		2	35.431.066	18.417.409	1,92378124	187,293422							
		4	20.048.581	10.323.409	1,94205044	189,072055							
		6	10.376.974	22.408.045	0,46309145	45,0851583							
	E3	0	44.703.936	29.200.865	1,53091136	100							
		2	42.963.622	18.302.137	2,34746478	153,337733							
		4	50.507.309	36.856.865	1,37036368	89,5129339							
		6	50.005.116	20.055.158	2,49337931	162,868953							
p300 + DMSC	E1	0	37.744.501	42.733.986	0,88324316	100		E1	E2	E3	E4	MV	STD
		2	31.382.551	36.420.744	0,86166694	97,5571591	0	100	100	100	100	100	0
		4	20.502.258	25.934.401	0,79054295	89,5045654	2	97,5571591	111,984848	94,8785027	145,398399	112,454727	23,2118324
		6	16.507.359	16.653.551	0,99122157	112,225219	4	89,5045654	99,0287142	168,237508	120,657321	119,357027	35,0970177
							6	112,225219	80,8005308	99,4111907	101,147887	98,3962067	13,0313893
	E2	0	27.413.915	32.993.693	0,8308835	100							
		2	37.830.279	40.657.451	0,93046362	111,984848							
		4	30.094.865	36.575.572	0,82281324	99,0287142							
		6	23.920.208	35.629.572	0,67135828	80,8005308							
	E3	0	52.853.915	48.024.894	1,10055246	100							
		2	39.122.693	37.467.108	1,04418769	94,8785027							
		4	55.092.794	29.755.087	1,85154202	168,237508							
		6	45.250.815	41.359.986	1,0940723	99,4111907							
	E4	0	33.454.915	43.579.229	0,76768029	100							
		2	38.881.087	34.833.602	1,11619485	145,398399							
		4	26.941.622	29.086.380	0,92626246	120,657321							
		6	22.523.915	29.007.258	0,77649239	101,147887							
p300 + EPO	E1	0	41.356.309	41.581.986	0,99457272	100		E1	E2	E3	E4	MV	STD
		2	31.496.137	40.437.693	0,77888066	78,3130929	0	100	100	100		100	0
		4	8.927.459	23.111.744	0,38627371	38,838156	2	78,3130929	111,721449	111,907202		100,647248	19,3421688
		6	11.827.803	45.172.602	0,26183577	26,3264579	4	38,838156	166,479262	90,9527624		98,7567267	64,1774053
							6	26,3264579	101,202805	89,0364002		72,1885543	40,1808943
	E2	0	40.595.844	48.794.886	0,83196924	100							
		2	36.491.258	39.259.522	0,92948809	111,721449							
		4	32.399.622	23.392.279	1,38505624	166,479262							
		6	28.434.673	33.771.350	0,8419762	101,202805							
	E3	0	32.401.480	27.891.137	1,16171241	100							
		2	47.581.087	36.599.714	1,30003986	111,907202							
		4	37.861.037	35.832.572	1,05660953	90,9527624							
		6	36.906.016	35.680.501	1,03434691	89,0364002							
