# Supplementary material for: TRIM25 targets p300 for degradation
Source: Life Sci Alliance. 2023 Sep 28;6(12):e202301980. doi: 10.26508/lsa.202301980 (PMC10539465; doi:10.26508/lsa.202301980)
Supplement: Supplementary file 8 [file LSA-2023-01980_SdataF4.pdf]

Figure 4A

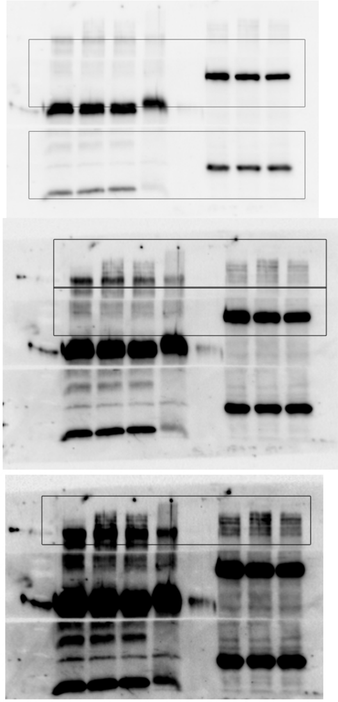

Figure 4B

Trim25-Wt					Trim25-Ko1			
dynein-p300	Dots	Nuclei	Dots/Nucleus		dynein-p300	Dots	Nuclei	Dots/Nucleus
	803	22	36,5			89	8	11,125
	288	4	72			10	14	0,71428571
	549	14	39,2142857			334	41	8,14634146
	391	27	14,4814815			128	12	10,6666667
	576	11	52,3636364			63	12	5,25
Total	2607	78	214,559404			624	87	35,9022938
		mean	42,9118807				mean	7,18045877
		STD	21,2045183				STD	4,30650802
		p	0,00610747					

Figure 4C

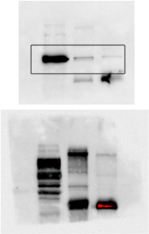

Figure 4D

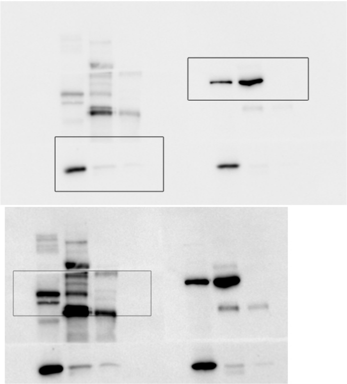

Figure 4E

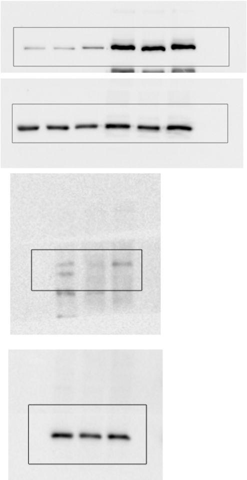

Figure 4F

Trim25-Wt					Trim25-Ko1				
a7-p300	Dots	Nuclei	Dots/Nucleus		a7-p300	Dots	Nuclei	Dots/Nucleus	
	372	34	10,9411765			39	42	0,92857143	
	346	32	10,8125			32	61	0,52459016	
	148	16	9,25			2	24	0,08333333	
	414	27	15,3333333			11	39	0,28205128	
	426	50	8,52			8	41	0,19512195	
	397	41	9,68292683			5	29	0,17241379	
Total	2103	200	64,5399366		Total	97	236	2,18608195	
			10,7566561	mean				0,36434699	mean
			2,425229	STD				0,31472013	STD
			1,0995E-06	p					
