# Supplementary material for: TRIM25 targets p300 for degradation
Source: Life Sci Alliance. 2023 Sep 28;6(12):e202301980. doi: 10.26508/lsa.202301980 (PMC10539465; doi:10.26508/lsa.202301980)
Supplement: Supplementary file 9 [file LSA-2023-01980_SdataFS6.pdf]

Figure S6A

WT-MCF7-DMSO		Exp3		
Dynein	Red dots	Nuclei	Dot/Cell	
1	855	45	19	
2	1141	50	22,82	
3	864	36	24	
4	768	41	18,73170732	
5	680	44	15,45454545	
6	970	46	21,08695652	
7	869	45	19,31111111	
8	358	19	18,84210526	
total	6505	326	159,2464257	
			**19,90580321**	Mean
			**2,676562339**	STD
		Dmso vs Epo:	0,00730764	p

WT-MCF7-Epoxo-7,5		Exp3		
Dynein	Red dots	Nuclei	Dot/Cell	
1	501	34	14,7352941	
2	422	24	17,5833333	
3	473	42	11,2619048	
4	361	31	11,6451613	
5	326	18	18,1111111	
6	301	36	8,36111111	
7	835	62	13,4677419	
8	201	18	11,1666667	
9	571	26	21,9615385	
10	239	13	18,3846154	
11	353	21	16,8095238	
	4583	325	163,488002	
		**325**	**14,8625456**	Mean
	**St.dev**		**4,07128089**	STD

Trim25-KO-1-MCF7- DMS(		Exp3	DMSO	
Dynein	Red dots	Nuclei	Dot/Cell	
1	178	19	9,36842105	
2	79	18	4,38888889	
3	81	19	4,26315789	
4	89	22	4,04545455	
5	130	29	4,48275862	
6	140	16	8,75	
7	299	22	13,5909091	
8	200	34	5,88235294	
9	132	26	5,07692308	
10	189	27	7	
11	138	23	6	
12	153	28	5,46428571	
13	166	32	5,1875	
	1974	315	83,5006518	
			**6,42312706**	Mean
			**2,81387484**	STD
	WTvs KO	9,59485E-10		

Trim25-KO-1-MCF7 -Epox(		Exp3	Epoxo	
Dynein	Red dots	Nuclei	Dot/Cell	
1	334	41	8,146341463	
2	128	12	10,66666667	
3	63	12	5,25	
4	99	10	9,9	
5	143	17	8,411764706	
6	38	6	6,333333333	
7	165	23	7,173913043	
8	287	52	5,519230769	
9	94	16	5,875	
10	183	27	6,777777778	
11	102	10	10,2	
12	137	17	8,058823529	
13	184	18	10,22222222	
14	203	24	8,458333333	
15	145	11	13,18181818	
16	96	17	5,647058824	
17	87	19	4,578947368	
18	187	12	15,58333333	
19	94	26	3,615384615	
	2769	370	153,5999492	
		**370**	**8,084207851**	Mean
	**St.dev**		**1,853963341**	STD
wt vs KO EPO		**p**	**1,57397E-05**	

Figure S6B

WT-MCF7-DMSO		Exp3		
a7	Red dots	Nuclei	Dot/Cell	
1	978	43	22,74418605	
2	452	34	13,29411765	
3	472	34	13,88235294	
4	416	33	12,60606061	
5	367	26	14,11538462	
6	369	18	20,5	
7	295	22	13,40909091	
8	631	37	17,05405405	
9	406	*28*	14,5	
10	555	28	19,82142857	
11	402	27	14,88888889	
Total	5343	330	176,8155643	
			**29,46926071**	Mean
			**3,441832795**	STD
		wtDMSO vs E	0,000322269	
		WT vs KO	2,52608E-05	
		wt EPO vs KC	p	1,68456E-09

WT-MCF7-Epoxo-7,5		Exp3		
a7	Red dots	Nuclei	Dot/Cell	
1	832	24	34,6666667	
2	1045	38	27,5	
3	1156	37	31,2432432	
4	457	25	18,28	
5	120	6	20	
6	561	30	18,7	
7	732	28	26,1428571	
8	908	35	25,9428571	
9	433	23	18,826087	
10	519	27	19,2222222	
11	703	21	33,4761905	
12	402	16	25,125	
	7868	310	299,125124	
			**46,0192498**	Mean
			**5,98493257**	STD

Trim25-KO-1-MCF7		Exp3		
a7	Red dots	Nuclei	Dot/Cell	
1	323	42	7,69047619	
2	197	13	15,1538462	
3	84	8	10,5	
4	145	18	8,05555556	
5	152	13	11,6923077	
6	141	11	12,8181818	
7	165	16	10,3125	
8	187	14	13,3571429	
9	175	18	9,72222222	
10	132	16	8,25	
11	143	16	8,9375	
12	371	40	9,275	
13	254	28	9,07142857	
14	205	14	14,6428571	
15	371	36	10,3055556	
16	156	14	11,1428571	
17	174	19	9,15789474	
	3375	336	180,085326	
			**10,5932544**	Mean
			**2,33794509**	STD

Trim25-KO-1-MCF7 -Epox(		Exp3		
a7	Red dots	Nuclei	Dot/Cell	
1	87	9	9,666666667	
2	49	4	12,25	
3	65	10	6,5	
4	99	10	9,9	
5	115	27	4,259259259	
6	98	13	7,538461538	
7	111	6	18,5	
8	126	13	9,692307692	
9	134	15	8,933333333	
10	149	7	21,28571429	
11	56	7	8	
12	467	38	12,28947368	
13	134	16	8,375	
14	194	23	8,434782609	
15	203	18	11,27777778	
16	183	15	12,2	
17	154	12	12,83333333	
18	186	17	10,94117647	
19	196	19	10,31578947	
20	162	13	12,46153846	
21	142	17	8,352941176	
	3110	309	224,0075558	
			**20,36432325**	Mean
			**45,63410406**	STD
	KO DMDO vs	p	0,944008388	
